# Follicular Helper T Cells: Potential Interventional Targets in Atherosclerosis

**DOI:** 10.1155/jimr/9247816

**Published:** 2025-10-13

**Authors:** Yuxuan Chen, Wenxin Wang, Xueli Xia, Xun Xu, Shengjun Wang, Poorani Gurumallesh

**Affiliations:** ^1^ Department of Laboratory Medicine, Jiangsu Province Engineering Research Center for Precise Diagnosis and Treatment of Inflammatory Diseases, Affiliated Hospital of Jiangsu University, Zhenjiang, China, ujs.edu.cn; ^2^ Department of Immunology, Jiangsu Key Laboratory of Laboratory Medicine, School of Medicine, Jiangsu University, Zhenjiang, China, ujs.edu.cn; ^3^ Institute of Active Polymers and Berlin-Brandenburg Center for Regenerative Therapies, Helmholtz-Zentrum Hereon, Teltow, Germany; ^4^ Institute of Chemistry and Biochemistry, Free University of Berlin, Berlin, Germany, fu-berlin.de

**Keywords:** atherosclerosis, cardiovascular disease, coronary artery disease, follicular helper T cell, germinal center, interventional targets

## Abstract

Atherosclerosis (AS) is a disease characterized by the presence of lesions in the arterial intima throughout the circulatory system. Lipid metabolism disorders form the pathological basis of AS. Immune injury resulting from lipid deposition, which is regulated by various cytokines, significantly contributes to disease progression. Follicular helper T (Tfh) cells are essential in the humoral immune response. The abnormal expression of surface molecules and cytokines by Tfh cells may contribute to the onset and progression of AS. Additionally, an increase in the Tfh cell population contributes to the progression of AS and coronary artery disease (CAD). Therefore, the targeting of Tfh cells and their associated functional molecules could serve as a promising therapeutic strategy against AS. This review summarizes current insights into the role of Tfh cells in AS and highlights their potential as therapeutic targets.

## 1. Introduction

Cardiovascular disease (CVD) is a leading cause of death and disability worldwide, with atherosclerosis (AS) being involved as a central underlying pathology. Vascular endothelial dysfunction, which is characterized by increased vascular permeability and inflammation, significantly contributes to early atherosclerotic lesion formation [1]. AS is a chronic inflammatory disease characterized by lipid deposition and plaque formation, and it predominantly affects large‐ and medium‐sized arteries. Moreover, it is the major contributor to CVD and hypertension and is associated with high morbidity and mortality rates [2, 3]. During AS, cholesterol, fat, calcium, and other substances accumulate beneath the endothelial lining of the coronary arteries, which gradually results in the formation and enlargement of plaques that narrow arterial lumens [4]. The deposition of oxidized LDL (oxLDL) in the arterial walls promotes coronary artery stenosis and triggers coronary artery disease (CAD). Associated inflammation further accelerates plaque development [4]. In rheumatoid arthritis (RA) patients, acute cardiovascular events are closely associated with rapid AS progression [5–8]. Bioinformatics analyses have revealed elevated levels of follicular helper T cell (Tfh), memory B cells, and γδT cells in both RA and AS, thereby indicating common inflammatory mechanisms [9]. Therefore, an increased focus has been applied to understanding the interaction between local and systemic inflammation and traditional cardiovascular risk factors in promoting AS progression.

Both humans and mice with AS develop IgG and IgM antibodies against oxLDL, with higher IgG levels being correlated with more severe disease and elevated IgM levels being associated with disease protection [10]. Tfh cells are a group of independent CD4^+^ T cell subsets that express chemokine receptor 5 (CXCR5), programmed cell death protein 1 (PD‐1), inducible costimulator (ICOS) and CD40 ligand (CD40L). Key transcription factors, such as achaete‐scute homolog 2 (Ascl2), T cell factor 1 (TCF‐1), lymphoid enhancer‐binding factor 1, and interferon regulatory factor 4 (IRF4) significantly regulate Tfh cell function [11–14]. Tfh cells drive AS progression by supporting B cell activation, anti‐LDL antibody production and germinal center (GC) formation [9, 10, 15–22]. In Bcl6‐/‐CD4^-^Cre^+^Ldlr‐/‐ mice, which lack functional Tfh cells, AS development was observed to be reduced by 20% to 30% [15]. In addition, Tfh cell‐mediated formation of aortic tertiary lymphoid organs (ATLOs) influences AS progression [19]. The plasticity and heterogeneity of Tfh cells are attributed to their spatial, environmental, and functional diversity. In addition to secondary lymphoid organs, Tfh cells accumulate in paravascular tertiary lymphoid organs (TLOs), in ATLOs and within atherosclerotic plaques and the vessel wall [17, 23, 24]. Current therapies (including statins and other lipid‐lowering agents) primarily focus on endothelial function, with little impact being observed on ectopic lymphoid tissue activity or adaptive immunity [25–28]. Therefore, recent studies have investigated the mechanisms, phenotypes and migratory behavior of Tfh cells in AS and CAD.

**Figure 1:**
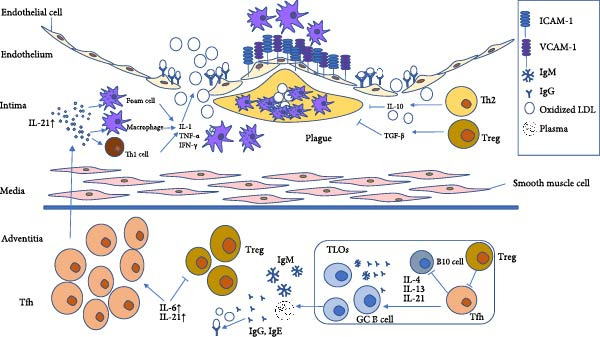
Activated Tfh cells are involved in the mechanism of aortic AS.

In this review, we synthesize these findings and explore combination strategies that target local Tfh cell‐mediated immune responses to mitigate plaque development and prevent cardiovascular events (Figure 1).

Within tertiary lymphoid structures in atherosclerotic arteries, Tfh cells help B cells to produce anti‐LDL antibodies, which form immune complexes with LDL. These complexes accumulate within the endothelial layer, thereby accelerating the disruption of the endothelial barrier and subsequently recruiting additional macrophages and platelets to promote local plaque formation. During this process, IL‐10 and TGF‐β (secreted by Th2 and Treg cells) contribute to the attenuation of plaque development. Moreover, adventitial Tfh cells secrete more IL‐21 into the intima, thereby activating macrophages and Th1 cells. Elevated levels of IL‐1, interferon (IFN)‐γ, and tumor necrosis factor (TNF)‐α cause a widening of endothelial cell (EC) junctions and upregulate ICAM‐1, VCAM‐1, and selectins, thus promoting monocyte and T cell recruitment, as well as endothelial infiltration. Recruited monocytes differentiate into macrophages, engulf excess oxLDL, and transform into foam cells. Foam cells, oxLDL, and inflammatory cytokines collectively drive plaque formation.

## 2. Tfh Cells in AS

### 2.1. Functional Molecules of Tfh Cells

T lymphocytes derived from the thymus mediated cellular immunity. T cells can be usually categorized into CD4^+^ helper T cells (including Th1, Th2, Th17, and Tfh cells), CD8^+^ cytotoxic T lymphocytes (CTLs), and regulatory T cells (Tregs) [29, 30]. It is well established that Tfh cells deliver key signals (via ICOS, CD40L, IL‐4, IL‐21, and signaling lymphocytic activation molecule [SLAM]‐associated protein [SAP]) to support B cell maturation, differentiation, survival, and antibody production [31–33]. ICOS is critically involved in Tfh cell initiation and migration into germinal centers (GCs) [34, 35]. Additionally, IL‐21 levels are positively correlated with Tfh cells, plasma cells, autoantibody production, and disease activity in patients with systemic lupus erythematosus (SLE) or RA [36]; moreover, IL‐21 sustains CD8^+^ T cell responses [37, 38]. The levels of IL‐21, as well as those of other inflammatory cytokines (such as IL‐6 and IL‐8), are reduced if ICOS is blocked [37, 39].

Peli1, which is an E3 ubiquitin ligase, restrains Tfh cell differentiation and activation by downregulating ICOS via the suppression of Krüppel‐like factor 2 (Klf2, which is a competitive inhibitor of the PI3K‐AKT pathway), thereby defining a Peli1‐ICOS‐Klf2 regulatory axis in autoimmune settings [40–45]. Its activation requires calcium flux (which is impaired in Bcl6‐deficient Tfh cells), thus hindering CD40L externalization and weakening Tfh‐B cell interactions, whereas CD40L itself remodels GC B cell morphology and motility to enhance Tfh cell engagement [46]. Moreover, CD40L alters the morphology and motility of GC B cells, thereby enhancing their interactions with Tfh cells. PD‐1, which is traditionally considered to be an inhibitory molecule on Tfh cells, also facilitates Tfh cell localization at the T‐B border and promotes their accumulation in GCs, thereby ensuring stringent GC selection [47]. PD‐1 antagonizes follicular (FOB) recruitment, and decreased CXCR5 expression leads to reduced PD‐1 levels [48]. Notably, the FOB homing defect of ICOS‐deficient T cells can be only partially restored by blocking bystander PD‐1/PD‐L1 interactions [47]. Recent evidence has enhanced our understanding of Tfh cell spatial heterogeneity. Specifically, CD90 and S1pr2 dictate GC‐Tfh cell fate, TIGIT directs Tfh cell differentiation potential, and CXCR4 regulates the niche distribution of these cells within lymphoid tissues [49–52].

### 2.2. Tfh Cells in the Development of ATLOs

B cells from the circulation enter peripheral immune organs through high endothelial venules (HEVs). At T–B borders, antigen‐specific B cells engage helper T cells that recognize the same antigen, thus facilitating their entry into the lymph nodes, where they proliferate, differentiate and form GCs. In AS, ATLOs develop in the aortic adventitia and expand over time (unlike the decreasing T cell populations in plaques) and are associated with disease progression [53]. In humans with AS, TLO size is positively correlated with plaque size and lesion instability [53, 54]. Tfh cells are critical for ATLO maintenance; specifically, a 2015 study revealed that Tfh cells promote B cell clustering in B220^+^ ATLOs and that anti‐ICOS blockade disrupts this architecture [19]. Moreover, CD3^+^CXCR5^+^ Tfh cells have been identified in the ATLOs of advanced AS patients and in abdominal aortic aneurysm lesions; additionally, a meta‐analysis confirmed that vascular‐associated SLOs rely on Tfh cells for their formation [17, 19]. Similar to secondary lymphoid organs, ATLOs contain lymph vessels and HEVs that facilitate the recruitment and migration of immune cells [55]. ATLO development is generally categorized into three stages. In the first stage, the T cell and B cell compartments are undifferentiated, with basal cells exhibiting high expression of CXCR13 and CCL21 [56–58]. Once the T cell and B cell compartments are separated, ATLOs progress to the next stage [58, 59]. In the final stage, ATLOs are well structured and contain activated GCs, lymph vessels, and FOB dendritic cells, which are considered to be the defining hallmarks of this specific stage [60].

### 2.3. Distribution of Tfh Cells in Lymph Nodes

Para‐aortic lymph nodes (PLNs) are located between the ascending aorta and the lateral aortic arch and span from the upper to the lower edge of the aortic arch. Tfh cell accumulation in PLNs is significantly greater compared with wild‐type (WT) mouse PLNs, which span from the ascending aorta to the lateral aortic arch. Compared with WT controls, PLNs exhibit marked Tfhcell accumulation and enlarged GCs in AS [16, 61, 62]. Moreover, Tfh cells aid GC B cells in producing antibodies, such as IgG1 and IgG3, which contribute to inflammation in AS [16]. However, the transfer of STAT4‐deficient Ldlr‐/‐CD4^+^ regulatory T (Treg) cells suppresses both GC responses and antibody titers. Dyslipidemia further amplifies autoimmune Tfh cell activation and IL‐27‐dependent IgG2c production while inhibiting follicular regulatory T (Tfr) cell differentiation, thereby exacerbating AS [15, 61]. Although Tfh cells are also present in mesenteric, inguinal and other lymph nodes, their site‐specific functions remain to be elucidated [16, 63, 64].

Lymphatic vessels in the aortic adventitia of ApoE‐/‐ mice facilitate reverse cholesterol transport and attenuate plaque development [65, 66]. Within GCs, Tfh cells are the predominant source of IL‐4, which inhibits lymphangiogenesis. Moreover, compared with ApoE‐/‐ control mice, ApoE‐/‐IL‐4‐/‐ mice exhibit a reduced plaque size due to enhanced lymphatic growth [67, 68]. Bcl‐6 inhibitors suppress CD4^+^IL‐4^+^ T cells but not Th2 cells, thus resembling the effects observed after the adoptive transfer of Tfr cells [64]. The ATP‐binding cassette transporter ABCA1, which is involved in reverse cholesterol transport, regulates cholesterol efflux; moreover, its mRNA is upregulated by the depletion of Tfh cells and Tfr cells, as well as being downregulated by Tfr cell transfer [64]. Thus, we propose that Tfh cells may inhibit lymphangiogenesis via IL‐4 secretion and contribute to the regulation of plasma cholesterol levels in LNs. However, the other roles of Tfh cells in lymph nodes remain poorly understood and warrant further investigation.

### 2.4. Enrichment of Tfh Cells in Plaques

Clinical analysis has demonstrated that Tfh cells accumulate in plaques [23]. Consistent with these findings, IL‐27 levels are elevated in plaques, thereby promoting the expression of IL‐6 and CXCL10, which attract infiltrating cells into endothelial cells (ECs) [16]. Disease progression is associated with continuous oxLDL infiltration into arterial ECs, which downregulates Treg cells and promotes Tfh cell proliferation [15]. In humans, IL‐6 mRNA expression in plaques is 10–40 times higher than that in normal plaques [69].

### 2.5. Distribution of Tfh Cells in the Aorta

Tfh cells induced by atheroma are present in AAA tissues in humans, where they express high levels of IL‐21, c‐MAF, and CXCL13, with these genes being observed to increase CVD risk [24, 70]. Tfh cells stabilize and promote the development of GCs in ATLOs, which contain B220^+^ B cell follicles with advanced atherosclerotic lesions in mice [19, 71]. The loss of CD8^+^ regulatory T cell (Treg) control or STAT4 deficiency alters this balance; specifically, without CD8^+^ Treg regulation, GCs in the adventitia expand and recruit peripheral B cells into the vessel wall, whereas STAT4 deficiency reduces adventitial Tfh cell numbers, elevates TGF‐β and suppresses Tfh responses [16, 19]. GC expansion is positively correlated with plaque growth [56], and Tfh cell‐derived IL‐4 and IL‐21 increase local IgG1, IgG4 and total immunoglobulin production, thereby further promoting adventitial inflammation and lesion progression [19].

### 2.6. Important Role of GCs in AS

**Figure 2:**
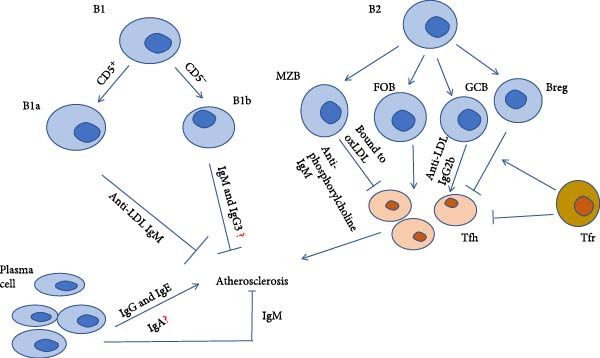
Role of B cells in AS.B1 cells are involved in innate immunity. B1a cells inhibit AS progression by secreting IgM antibodies against LDL. B1b cells also secrete IgM and IgG3 against LDL; however, their role in AS remains unclear. B2 cells are involved in adaptive immunity. MZB cells have garnered increasing research attention because of their antiatherosclerotic activity, which includes the secretion of antiphosphorylcholine IgM and direct binding to oxLDL. In contrast, FOB cells promote GCB formation, thus leading to the production of anti‐LDL IgG, which accelerates disease progression. “?” = uncertain effects.

Different B cell subsets exert distinct effects on AS. For example, IgM secreted by B1 cells is thought to protect against AS, whereas IgG from B2 cells tends to be pathogenic [62, 72, 73]. A recent study revealed that plasma cells can increase plaque size [62], thus suggesting that IgM may not play a central role in AS. Although antibody deficiency may alleviate the disease, it also reduces plaque stability, thereby leading to plaque rupture [74]. The two major B2 cell subsets known as FOB and marginal zone B (MZB) cells regulate Tfh cells in AS via distinct pathways. Specifically, adaptive FOB cells promote AS by supporting Tfh cell and GC responses, whereas MZB cells protect against AS by regulating the process in a T cell‐independent manner (Figure 2) [19, 75]. The IgG2b concentration is partially dependent on splenic GCs in vivo, particularly regarding specific antibodies derived from GCs that recognize modified LDL [62]. B10 cells and plasmablasts secrete IL‐10, thereby alleviating the severity of AS in the Ldlr‐/‐ model [76]. Moreover, immune complexes promote smooth muscle cell (SMC) proliferation, which contributes to plaque formation [72, 77]. Genetic defects in GCs suppress SMC proliferation by inducing metabolic dysregulation, thus reducing the plaque burden [62]. These findings align with the results of previous studies demonstrating an increased ratio of GC B cells to total B cells in ApoE‐/‐ mice [19, 75]. These mice also exhibited an enrichment of GC‐like cells in the aorta and secondary lymphoid organs compared with IL21^–/–^ mice [63]. Furthermore, FCγRIIb in GC B cells is negatively correlated with the AS response, due to the fact that FCγRIIb limits GC expansion and regulates IgG2a and IgG2c class switching, which can promote macrophage or DC responses [78–81].

### 2.7. Pathogenic Role of IL‐21 in AS

IL‐21, which is the primary cytokine produced by Tfh cells, regulates the proliferation, maturation, and function of immune cells, including B cells, T cells, and NK cells. An understanding of the regulation of IL‐21 is essential for evaluating its impact on disease.

Tfh cells have been observed to be proatherogenic [15, 19]. In IL‐21‐deficient mice, Ang II infusion selectively reduces PD‐1^+^CXCR5^+^CD4^+^ Tfh cells without changing total CD4^+^ T cell frequencies, thereby resulting in a relative increase in PD‐1^-^CXCR5^+^ non‐Tfh cells [63]. Compared with WT controls, Bcl6^fl/fl^CD4^cre^ mice, which lack functional Tfh cells, exhibit similar GC defects and protection from Ang II‐induced hypertension, including lower IgG2c levels [15]. Overall, these data implicate IL‐21‐dependent GC reactions in the regulation of blood pressure. In AAA, Tfh cells secrete significantly more IL‐21 compared with non‐Tfh cells [19], which aligns with the association of AS with IL‐21‐dependent GC reactions. These results suggest that IL‐21 plays a pathogenic role in AS development, which is largely dependent on Tfh cells. The involvement of Th cells is also evident.

IL‐21 directly downregulates NO secretion by ECs, thereby impairing endothelium‐dependent vasodilation and promoting endothelial dysfunction [63]. Additionally, IL‐21 regulates the ratio of Th1, Th17, and Treg cells, thus further influencing the expression of IL‐17 and IFN‐γ [82–85]. IL‐21 secreted by Tfh cells upregulates genes associated with IFN‐γ production, thereby enhancing the Th1 response, which is further aggravated by IL‐15 or IL‐18 secretion [86, 87].

Cytokine‐induced activation of ECs leads to endothelial dysfunction, thereby upregulating the expression of adhesion molecules and chemokines, which promotes immune cell migration to atherosclerotic sites. Cytokines exacerbate this process [88, 89]. Moreover, IFN‐γ and TNF‐α (which have been demonstrated as being proatherogenic cytokines) induce cytoskeletal reorganization, thereby increasing the gaps between ECs [90]. IFN‐γ deficiency alleviates AS in ApoE‐/‐ and Ldlr‐/‐ mice [91–94]. In contrast, IL‐1RA and IL‐33 inhibit AS by regulating macrophage cholesterol handling [89]. Additionally, IL‐21 induces Th1 cell enrichment by increasing IFN‐γ expression, which triggers positive autocrine feedback, thus amplifying and stabilizing IL‐21‐driven, T cell‐mediated responses. IL‐21 also induces neutrophil infiltration, which can damage cardiomyocytes and promote inflammation in ECs, thereby leading to increased LDL deposition [95, 96].

In IL‐21‐deficient (IL‐21‐/‐) mice, a high‐fat diet exacerbates insulin resistance, thus underscoring the role of IL‐21 in metabolic homeostasis. Mechanistically, IL‐21 suppresses IRF4 expression, thereby downregulating obesity‐associated inflammatory mediators; this inverse relationship between IL‐21 levels and lipolysis has also been documented in obese humans. Taken together, these data suggest that reduced IL‐21 activity promotes fat accumulation at arterial inflammatory sites [97].

In CVD patients, coronary heart disease is a chronic inflammatory condition that often leads to the formation of collateral vessels in chronic coronary artery occlusion [98]. Blood flow disruption increases LDL permeability, thus causing LDL retention in the gaps between ECs [99]. Additionally, IL‐21 and its receptors are abnormally expressed in cardiovascular and cerebrovascular disease patients [100]. In combination with IL‐15, IL‐21 increases T‐bet mRNA levels, thus amplifying the Th1 response and accelerating disease progression [101]. In patient serum, IL‐21 expression is higher than that in healthy individuals and is closely correlated with total cholesterol and LDL cholesterol levels [102]. These findings are consistent with increased IL‐21R expression in the muscle ECs of peripheral artery disease patients, where the angiogenic effect is regulated by STAT3 signaling activation under ischemic conditions [103].

### 2.8. Circulating Tfh (cTfh) Cells in CAD Patients

CAD arises from AS; specifically, when plaque‐induced stenosis exceeds 50% in the coronary arteries, myocardial ischemia occurs. Among CD4^+^ T cells, cTfh cells (CD4^+^CXCR5^+^) in the peripheral blood functionally mirror lymphoid Tfh cells. Moreover, cTfh cells express CXCR5, ICOS, PD‐1, and Bcl6; additionally, they exhibit reaction kinetics and transcriptional profiles analogous to those of lymphoid Tfh cells [104–107], due to these similarities, cTfh cells provide a peripheral surrogate for the investigation of lymphoid Tfh‐driven humoral responses [107].

Peripheral cTfh cells are the most potent subset for inducing B cell class switching and antibody production in humans [104]. In cocultures with cTfh cells, B cells from coronary heart disease patients exhibit increased STAT1/STAT4 phosphorylation and upregulated IL‐6 and IFN‐γ gene expression. Moreover, CAD patients have elevated frequencies of CD4^+^CXCR5^+^PD‐1^+^CCR7^-^ cTfh cells, which more effectively support mature antibody secretion and proinflammatory responses [108–110].

Overall, cTfh cells (CD4^+^CXCR5^+^ICOS^+^) are broadly classified into three subsets (cTfh1, cTfh2, and cTfh17 cells) based on CXCR3 and CCR6 expression; however, alternative classification schemes have also been proposed (Table 1) [104, 111]. Moreover, cTfh1, cTfh2, and cTfh17 cells are characterized by the expression of distinct transcription factors and secretion of various cytokines. Specifically, cTfh1 cells express T‐bet and produce IFN‐γ; cTfh2 cells express GATA3 and release IL‐4, IL‐5, and IL‐13; and cTfh17 cells express RORγT and secrete IL‐17A and IL‐22 [104, 110, 112]. The cTfh2 and cTfh17 subsets function as efficient B cell helpers, which is primarily achieved via IL‐21 production (a capacity that is often elevated in autoimmune diseases) [112]. In contrast, cTfh1 cells are less effective at supporting antibody responses [110]. Imbalances among these subsets are implicated in the pathogenesis of autoimmune and inflammatory disorders [104, 113–115]. However, the distribution and functional roles of distinct cTfh subsets in AS remain largely unexplored. Compared with healthy individuals, cTfh cells from CAD patients induce enhanced phosphorylation of STAT1 and STAT3 in B cells, and increase the secretion of IL‐6 and interferon‐γ by B cells [21]. The population of cTfh cells is observed to be increased in the peripheral blood of CAD patients [21, 116]. Additionally, CAD patients demonstrate elevated serum levels of IFN‐γ, IL‐17A, and IL‐21; moreover, cTfh cells secrete these cytokines more robustly compared with non‐Tfh cells [108]. In various autoimmune diseases, cTfh17 cells are significantly increased and positively correlated with disease progression and autoantibody titers [104, 113, 117]. In CAD patients, cTfh17 cells are markedly expanded and most potently promote B cell antibody secretion, of which the reduced ability to secrete IL‐10 further promotes this process [21]. Additionally, their frequency is correlated with disease severity and constitutes an independent risk factor for CAD [21, 104]. Prior to this, IL‐17 had been found to promote AS in mice [118–120]. The proportion of cTfh1 cells is increased in CAD patients and, together with cTfh cells, contributes to the progression of CAD [21, 108]. In contrast to the effect of cTfh17, cTfh2 appears to play a protective role in CAD patients. However, the proportion of cTfh2 is reduced in these patients, along with decreased production of IL‐10, IL‐4, and IL‐5, which weakens their atheroprotective function [21, 108, 121]. In AS, the excessive expansion of cTfh1 and cTfh17 cells, as well as the reduction in cTfh2 cells, lead to plasmablast accumulation, B10 cell suppression, and elevated serum IgM, IgG, and IgA levels, thus reflecting persistent cTfh cell imbalance and cTfh17 cell skewing; however, the underlying mechanisms of these effects require further investigation [21].

**Table 1:** Classification of cTfh subsets in CAD.

cTfh subsets	Phenotype	Production	Effects	Cause	References
cTfh1↑	CD4^+^CXCR5^+^ICOS^+^CXCR3^+^CXCR6^-^	IFN‐γ↑	Upregulate plasmablasts and downregulate B10 cells	Increased LDL; metabolic and inflammatory disorders	[21, 104]
cTfh2	CD4^+^CXCR5^+^ICOS^+^CXCR3^-^CXCR6^-^	IL‐4↓, IL‐5↓, IL‐13	Elevate levels of anti‐LDL antibodies	Dysregulated cTfh development	[21, 104]
cTfh17↑	CD4^+^CXCR5^+^ICOS^+^CXCR3^-^CXCR6^+^	IL‐17↑	Upregulate plasmablasts and downregulate B10 cells	Increased LDL; metabolic and inflammatory disorders	[21, 104]
Circulating precursor Tfh cells	CD4^+^CXCR5^+^ICOS^+^CCR7^hi^PD1^lo^CD45RA^lo^	—	Resting conditions	—	[108, 109]
	CD4^+^CXCR5^+^ICOS^+^CCR7^lo^PD‐1^hi^ CD45RA^lo^	IFN‐γ, IL‐17 A, IL‐21	Induce plasmablast differentiation, induce antibody secretion, increase the expression of IFN‐γ and IL‐6	Total cholesterol and LDL levels	[108, 109]

Due to the fact that cTfh17 cells are critical in CAD, cTfh subset proportions depend on the cTfh1:cTfh17 ratio; moreover, Ding et al. reported that blood oxLDL levels directly regulate Tfh1 and Tfh17 frequencies, thus potentially driving subset imbalance [21].

### 2.9. Potential Factors Promoting Tfh Cells in AS

A recent study revealed that STAT4 deficiency in LDLr‐/‐ mice increased the number of CD8+ Tregs, thereby reducing the Tfh cell frequency in both the spleen and aortic arch compared with that in STAT4^+^LDLr‐/‐mice [16]. However, the Qa‐1 mutation, which disrupts Treg cell function, induces excessive activation of the Tfh‐GC B cell axis, thereby promoting AS development [19]. The loss of Foxp3 expression deprives Treg cells of their immunosuppressive function, thus allowing their conversion into Tfh cells, which accelerates AS [61].

Both innate and adaptive immunity play crucial roles in AS, with many traditional risk factors being linked to changes in arterial function. As innate immune cells, macrophages play a significant role in promoting AS. Under STAT4 control, macrophages can accelerate Tfh cell differentiation and exacerbate AS [16, 19]. Additionally, macrophages upregulate Treg cells and suppress Tfh cells via TGF‐β adjustment in STAT4‐LDLr‐/‐ mice [16]. This finding is consistent with findings demonstrating that TGF‐β induces Tfh cell differentiation through the STAT3/STAT4 pathway and that STAT4 (in combination with IL‐12) increases IL‐21 and Bcl6 expression, thereby supporting Tfh cell generation [122–126]. These results suggest that STAT4 plays a key role in the Tfh/CD8^+^ Treg cell axis, thus significantly increasing atherosclerotic plaque burden.

## 3. Potential Strategies for Targeting Tfh Cells in the Treatment of AS

### 3.1. The Targeting of Cytokines Associated With Tfh Cells

IL‐21 regulates the proliferation, maturation and effector functions of multiple hematopoietic lineages (including B cells, T cells, NK cells and dendritic cells) while also suppressing Foxp3 expression and thereby limiting Treg development [15]. In murine models, the blockade of IL‐21 prevents endothelial dysfunction, attenuates vascular inflammation and restores endothelium‐dependent vasodilation by normalizing proinflammatory Tfh cell levels in the aorta [63].

AS is closely related to chronic autoimmune diseases, such as SLE and RA [61, 127, 128]. Atherogenic dyslipidemia amplifies CXCR3^+^ Tfh cell responses and IgG2c production, thereby aggravating SLE, whereas the transcription factor isoform known as Pbx1d, which is highly expressed in Tfh cells, drives SLE pathogenesis in dyslipidemic mice [61, 127].

### 3.2. The Targeting of Surface Molecules Associated With Tfh Cells

PD‐1, which is a key molecule involved with Tfh cells, suppresses the functions of these cells. Research on the PD‐1/PD‐L1 pathway has demonstrated that IFN‐γ and TNF‐α upregulate PD‐L1 expression, which suppresses Tfh cells and CD8^+^ T cells [129]. The use of PD‐L1 blockers has revealed that PD‐1/PD‐L1 deficiency impairs vascular ECs by enhancing CD8+ T cell cytotoxicity and GC‐derived antibodies, thus contributing to early AS [130–132]. PD‐1/PD‐L1 blockade is used in cancer immune checkpoint therapy; however, this specific therapy may accelerate the progression of AS in patients with CAD [133–135]. Recent studies have demonstrated that, in vitro IFN‐γ‐stimulated B cell transfer inhibits Tfh cell activation and reduces AS in Ldlr‐/‐ mice, thus suggesting a novel treatment approach [136].

CD40L is essential for Tfh cell development; moreover, its deficiency impairs Tfh cell frequency and function [137]. The CD40/CD40L pathway activates B cells, T cells, and GCs, thereby increasing the expression of the atherogenic cytokines IL‐6, IFN‐γ, and TNF‐α [138–140]. The pathway also upregulates VCAM‐1 and P‐selectin on ECs and activates platelets, thus promoting thrombosis [141, 142]. Anti‐CD40 mAbs have demonstrated efficacy in phase 1 and phase 2 tumor trials and may be potential treatments for AS [143, 144].

ICOS is essential for Tfh cell activation, as well as for migration into and maintenance within GCs, via engagement with its ligand ICOSL (a B7‐family member expressed on B cells and antigen‐presenting cells that mediates ICOS‐dependent recruitment). Upon ICOSL binding, ICOS signaling downregulates Klf2 (unlike what occurs via PD‐1 signaling), thereby restraining further Tfh cell differentiation [42]. Therapeutically, ICOSL blockade with monoclonal antibodies in ApoE‐/‐ mice reduces the atherosclerotic burden by ~ 30%, which coincides with diminished total Tfh cell numbers (including ex‐Treg‐derived CD25^+^Foxp3^+^ cells) in the aorta and PLNs, as well as reduced IgG deposition at lesion sites, thereby demonstrating effective prevention of GC and Tfh cell expansion in AS [15, 19].

### 3.3. Regulation of the Ratio of Tfh Cells to Treg Cells

In vivo ApoAI expression reduces the Tfh cell frequency and maintains Treg cell levels [145, 146]. oxLDL induces plaque formation and alters IL‐2 and IL‐6 receptor expression on Treg cells, thereby impairing Treg cell homeostasis and increasing the generation of Tfh cells. The addition of ApoAI to Treg cells cultured with oxLDL in vitro reduces oxLDL‐induced Treg cell differentiation [15]. Under certain conditions, exTreg cells can be converted into Tfh cells, thereby disrupting the balance between Tfh cells and Treg cells due to downregulated STAT5 expression and upregulated IL‐6R expression [15]. Continuous ApoAI injections for 9 weeks have been observed to suppress Treg‐to‐exTreg cell conversion, thus reducing the atherosclerotic burden [15]. ApoAI maintains immune homeostasis in AS by preserving Treg stability and limiting proatherogenic Tfh cell responses. In vivo ApoAI expression (or continuous ApoAI injections for 9 weeks) sustains IL‐2Rα while downregulating IL‐6Rα and Bcl6, thereby preserving STAT5 signaling, preventing ex‐Treg‐to‐Tfh cell conversion and reducing plaque burden [15, 145–152]. Conversely, oxLDL drives plaque formation and destabilizes Treg homeostasis by downregulating IL‐2R and upregulating IL‐6R, thereby skewing differentiation toward Tfh cells both in vitro and in vivo [15, 97, 151, 152].

Tfh cells are proatherogenic and play a key role in GC responses to a high‐cholesterol diet (HCD) [19, 20]. Tfr cells counteract this effect by reducing the frequency of Tfh cells in ApoE‐/‐mice, rebalancing Th1/Th2 responses, and expanding regulatory B (Breg) cells via the PD‐L1 and TGF‐β pathways [20, 64, 153]. Tfr‐derived neuritin further inhibits IgE production, which increases IgM levels and decreases IgE levels in lesions; moreover, their reciprocal regulation with Bregs amplifies the suppression of Tfh cell responses [64, 154]. Moreover, Tfr cells promote lymphangiogenesis, thus facilitating reverse cholesterol transport and reducing local cholesterol accumulation [66, 155–157]. Although, Tfr cell transfer exhibits limited efficacy in established plaques, it represents a promising preventive strategy (particularly for familial hypercholesterolemia) by preventing early Tfh cell‐mediated GC expansion and lipid‐driven inflammation [20, 64, 158] (Table 2).

**Table 2:** Potential therapeutic strategies targeting Tfh cells in AS.

Therapeutic agent	Model	Mechanism of action	References
Anti‐IL‐21 mAb	Human (phase I safety)	IL‐21/IL‐21R blockade attenuates	[159]
Anti‐BAFFR mAb	ApoE‐/‐mouse	Tfh cell‐driven germinal center responses and endothelial dysfunction. BAFFR blockade induces B2 cell apoptosis, thus reducing GC formation and autoantibody titers.	[160]

Anti‐ICOS mAb	ApoE‐/‐mouse	ICOSL blockade reduces Tfh‐B cell interactions, germinal center formation, and aortic lesion size.	[15, 19, 64]

Anti‐CD40L mAb	Mouse	CD40L blockade inhibits B cell costimulation and downstream inflammatory cytokine release.	[143, 144]
Anti‐CD20 mAb	Human and mouse	FcγR‐dependent B cell depletion diminishes Tfh‐dependent humoral responses.	[161]
IFN‐γ‐stimulated B cells	LDLr‐/‐mouse	PD‐L1 upregulation on B cells suppresses Tfh cell activation and GC responses.	[136]

CD27 agonist	ApoE‐/‐mouse	CD27 costimulation expands regulatory T cells and limits Tfh cell proliferation.	[162]
Maresin‐1	Mouse	Maresin‐1 promotes Treg‐mediated resolution of inflammation and indirectly restrains Tfh cell‐driven GC activity.	[163]

Abbreviations: BAFFR, B cell‐activating factor receptor; mAb, monoclonal antibody.

## 4. Conclusion

AS is now regarded as being a chronic immune‐inflammatory disease driven by both innate and adaptive responses. Tfh cells (which are essential for B cell proliferation, differentiation, class‐switch recombination, antibody production, and GC formation) are dysregulated in AS and directly contribute to its progression. In patients with CAD, the numbers of circulating and tissue‐resident Tfh cells are increased, which is correlated with upregulated inflammatory gene expression and increased cytokine release. These findings suggest that effective therapies are needed to dampen pathogenic Tfh cell‐mediated humoral responses and modulate the local immune microenvironment to halt or reverse plaque development [15, 61, 164]. Compelling evidence links AS with autoimmune comorbidities, such as SLE and RA, thereby underscoring the complexity of its immune dysregulation. In both AS and CAD, GC‐driven responses exacerbate vascular injury, whereas strategies that reduce GC reactivity (whether by directly targeting Tfh cells or by modulating their microenvironment) can restore a healthier vascular milieu. However, our understanding of the precise mechanisms by which Tfh cells promote plaque formation remains incomplete. Therefore, this study sought to elucidate Tfh cell‐mediated adaptive immune pathways in AS and CAD, with the ultimate goal of developing combination immunotherapies that suppress pathogenic GC activity, halt disease progression and improve patient outcomes.

**Nomenclature**
IL: Interleukin
TNF: Tumor necrosis factor
IFN: Interferon
TLOs: Tertiary lymphoid organs
ICAM‐1: Intercellular cell adhesion molecule‐1
VCAM‐1: Vascular cell adhesion molecule‐1.

## Disclosure

All of the authors have read and approved the final version of the manuscript.

## Conflicts of Interest

The authors declare no conflicts of interest.

## Author Contributions

Yuxuan Chen drafted the manuscript. Wenxin Wang, Xueli Xia, and Xun Xu discussed and revised the manuscript. Shengjun Wang designed the study and revised the manuscript.

## Funding

The study was funded by the Jiangsu Province “333” Project (Grant BRA2017128), Jiangsu Provincial Medical Key Discipline Cultivation Unit (Grant JSDW202241), and the Postgraduate Research & Practice Innovation Program of Jiangsu Province (Grant KYCX24_4029).

## Data Availability

The data that support the findings of this study are available from the corresponding author upon reasonable request.
